# Age-period-cohort analysis on the cancer mortality in rural China: 1990–2010

**DOI:** 10.1186/1475-9276-13-1

**Published:** 2014-01-02

**Authors:** Peigang Wang, Chunling Xu, Chuanhua Yu

**Affiliations:** 1 School of Public Health, Global Health Institute, Wuhan University, Wuhan, China

## Abstract

**Background:**

Cancer has become a global health problem. China still suffers continuous increasing cancer mortality. To study the trend of cancer mortality in rural China, this paper established an Age-Period-Cohort model to discuss the age effect, period effect and cohort effect on cancer mortality in rural China.

**Methods:**

The data were collected from the “China Health Statistical Yearbook” from 1990 to 2010. Collected data were analyzed by Age-Period-Cohort model and Intrinsic Estimation method.

**Results:**

The age effect on the total cancer mortality represented a V trend. Compared with Group 0–4, Group 5–9 showed 71.87% lower cancer mortality risk. Compared with Group 5–9, Group 75–79 showed 38 times higher cancer mortality risk. The period effect on the total cancer mortality risk weakened firstly but then increased. It increased by 35.70% from 1990 to 2010, showing an annual average growth of 1.79%. The cohort effect on the total cancer mortality risk weakened by totally 84.94% from 1906–1910 to 2005–2010. Three “deterioration periods” and three “improvement periods” were witnessed during this period. The malignant cancer mortality varied similarly with the total cancer mortality, while benign cancer mortality and other cancer mortality represented different variation laws.

**Conclusions:**

Although the total cancer mortality risk is increasing at an accelerated rate, cancer mortality risk in recent born year is decreasing, indicating very important impact of social change on the cancer mortality in rural China.

## Introduction

Generally, cancer mortality is increasing continuously, and becomes the second leading cause of death in developed countries and third leading cause of death in developing countries [1]. It was estimated that there were 12.7 millions of new cancer cases and 7.6 millions of cancer deaths around the world in 2008. Among them, 56% new cases and 63% deaths were contributed by underdeveloped regions [2]. With the continuous increase and aging of world population, cancer mortality is expected to further increase [2,3]. Although cancer became a disease burden in developed regions firstly, cancer mortality in other places is expected to exceed that in developed regions in the future [4]. Cancer mortality in some underdeveloped or economic transitional regions such as Africa and Asia is still increasing [5]. It is estimated that in 2020, developing countries and emerging industrialized countries will suffer the quickest growth of cancer mortality, while some western countries will decrease cancer mortality as they adopted tobacco resistance and healthy lifestyle [1].

As a large population country in the world, China accounts for 1/4 of the total cancer deaths with quick increasing cancer mortality. This will absolutely bring great impact on the global variation trend of cancer and cancer burden, thus deserving deeper studies [6,7]. Some research compared the cancer mortality in developed countries and developing countries, reporting that cancer mortality in China has both characteristics of developed countries and developing countries [8]. According to the “2012 Annual Report of Cancer in China”, incidence rate, mortality risk and younger patient proportion of cancer all increased during the past two decades. It is estimated an annual increase of 3.12 millions of cancer cases and a daily increase of 8,850 cancer cases in China. Furthermore, there are 6 persons diagnosed as malignant cancer per minute. Furthermore, 22% of Chinese residents are at the risk of cancer [9,10]. It is predicted that the malignant cancer incidence and mortality in China will continue to increase in the next two decades [11]. Malignant cancer not only has become a serious disease that threatens people’s survival and social development in China, but also brings heavy burden for both families and the society [12].

With the increasing cancer burden in China, the different trend of cancer mortality in China has attracted increasing attentions from researchers. There are researches on selected cancer mortality for limited populations and time periods (e.g. lung cancer, nasopharyngeal cancer, stomach cancer, esophagus cancer, liver cancer, breast cancer and cervical cancer), and also researches on the cancer mortality in special regions (e.g. Shandong, Henan, Hebei, Taiwan and Hong Kong) [13-21]. Furthermore, there are three major researches focusing on the national cancer mortality [22,23]. However, they failed to give the cohort effect analysis or the whole cancer variation trend analysis in a long history perspective. To make a national plan of time-sensitive and purposeful cancer prevention planning as well as effect evaluation of existing measures, a latest comprehensive evaluation on cancer mortality is needed [24].

In this paper, the age effect, period effect and cohort effect were discussed by establishing an APC model. A statistical analysis on the cancer mortality of 0–84 year old from1990 to 2010 was carried out in rural China, involving total cancer mortality, malignant cancer mortality, benign cancer mortality and other cancer mortality. The period effect (since 1990) and cohort effect (since 1906) on cancer mortality were explored on the basis of age effect. Key attentions were paid to the analysis of cohort effect, including level and variation rate of cohort effect. The systematic analysis on the research results not only lays foundation for discussing the relationship between social development and cancer burden, but also can assist to explore the influence factors of cancer incidence and development. The research results can be served as scientific references for making national cancer prevention.

## Methods

### Data source and processing

Data were collected from the “China Health Statistical Yearbook” of 1990, 1995, 2000, 2005 and 2010. The mortality statistics of these five years enjoy high reliability and authority. Data of 2000 is not available, mean scores of 1999 and 2001 are used to substitute for it. Furthermore, mortality statistics were based on an age group of five years old. The APC model requires death statistics per five years. Among the involved data, age-specific cancer mortality took the cancer mortality in age statistics of the year/census population of the year × 1/100,000 as the proportionality coefficient. Originally, mortality statistics about 0 year old and 1–4 years old were independent from each other. However, these two age groups were integrated in this paper for Age-Period-Cohort (APC) model analysis (5-year a group). The last population census before 1990 was carried out in 1982, which can’t be input in APC model.

Only data from Group 0–4 to Group 80–84 were applied. Data above 85 years old in the “China Health Statistical Yearbook” were integrated into one age group which also involving those above 89 years old. Therefore, this age group couldn’t be used for APC model analysis.

### Statistical analysis of APC model

Since APC model can explore age effect, period effect and cohort effect from the observed age-specific death data, it is widely applied in sociological, demographical and epidemiologic studies [25]. In this paper, the latest Intrinsic Estimation (IE) algorithm in the APC model that had been confirmed as estimability, non-bias, validity and asymptotic was applied [25]. Currently, the IE algorithm has been used to study human mortality and disease incidence in developed countries and regions.

The APC model for statistical analysis was established on the basis of cancer mortality data in rural China from the “China Health Statistical Yearbook” of 1990, 1995, 2000, 2005 and 2010. The earliest cohort in our APC model was Group 80–84. They were born during 1906–1910. Therefore, the established APC model can estimate the cancer mortality of people born from 1906 to 2010.

In this paper, data were analyzed through the apc_ie that was specially designed for IE algorithm of APC model [25]. The data-model fitting degree was evaluated by fitting deviance, AIC and BIC. The standard error (SE) of every model coefficient was calculated. Additionally, odds ratio was calculated from estimated model parameters, which represented the mortality level. The mortality change was expressed by the difference of odds ratio before and after the birth cohort.

## Results

### The age, period and cohort-based variation of cancer mortality

#### Age-based variation of cancer mortality

**Figure 1:**
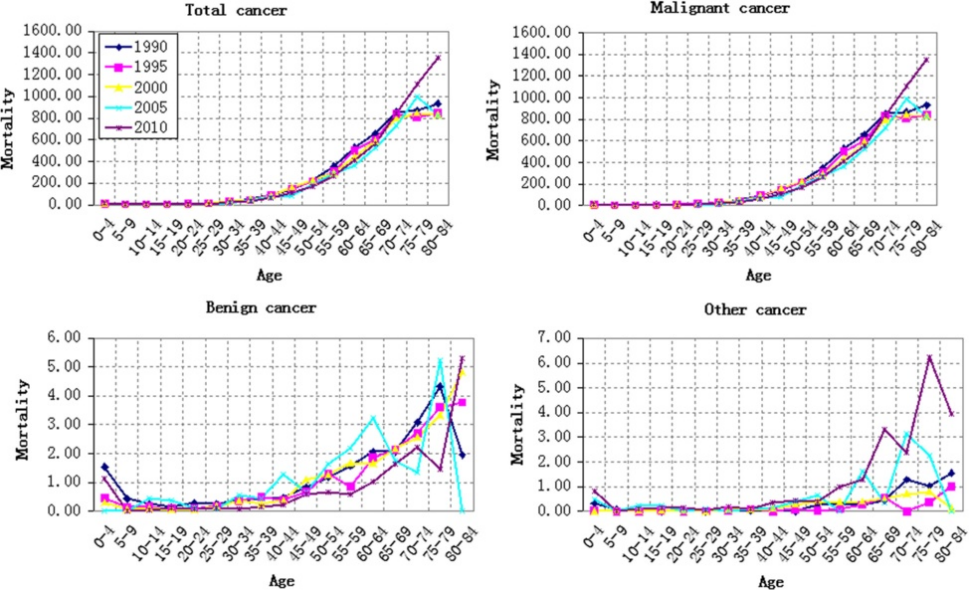
Age-specific cancer mortality in different years (per 100,000 persons).

The variation of cancer mortality in rural China in 1990, 1995, 2000, 2005 and 2010 with age was shown in Figure 1. Firstly, the total cancer mortality risk increased with age. Before 20 years old, the cancer mortality risk was lower without obvious change (Figure 1). Combined with the “China Health Statistical Yearbook”, it is easy to find that Group 0–4 suffers the highest cancer mortality risk, which fluctuated with ages. The cancer mortality risk increased at a steady speed after 20 years old. Before 75 years old, the increase of cancer mortality accelerated as growing elder. However, after 75 years old, the increase of cancer mortality risk in 1990 and 1995 slowed down and the slope of corresponding broken line decreased in Group 70–74 (Figure 1). The cancer mortality risk of Group 80–84 decreased in 2000 and 2005, quicker in 2005. However, the total cancer mortality in 2010 presented a sharp increase from 7.18/100,000 of Group 20–24 to 1356.30/100,000 of Group 80–84.

Malignant cancer mortality accounts for about 90% of the total cancer mortality in all years and among all age groups. This is why malignant cancer mortality varied similarly with total cancer mortality. The following statistical results, including period effect, cohort effect, APC model analysis results and change rate of cohort, also showed similar variation law. Therefore, the malignant cancer mortality wasn’t analyzed independently to every result.

Benign cancer mortality was relative lower, peaking at only (5.30/100,000) of Group 80–84 in 2010. It decreased from Group 0–9 and then fluctuated gently. However, it showed a wave growth after 35 years old and fluctuated more violently in older groups. Additionally, other cancer mortality was even lower except for some age groups, such as Group 75–79 in 2010 (6.25/100,000). The benign cancer mortality risk decreased from Group 0–9 and then its broken line became close to the X-axis. However, it increased significantly after 35 years old and showed a wave growth after 65 years old. The most violent fluctuations were in 2005 and 2010.

#### Period-based variation of cancer mortality

**Figure 2:**
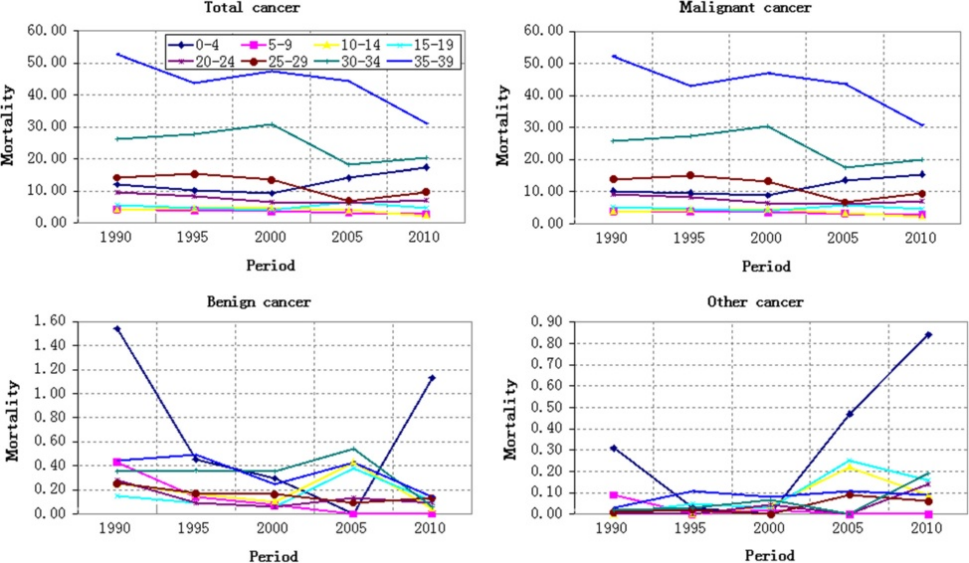
Cancer mortality (100,000 persons) variation of different age groups during 1990–2010: 0–39 years old.

**Figure 3:**
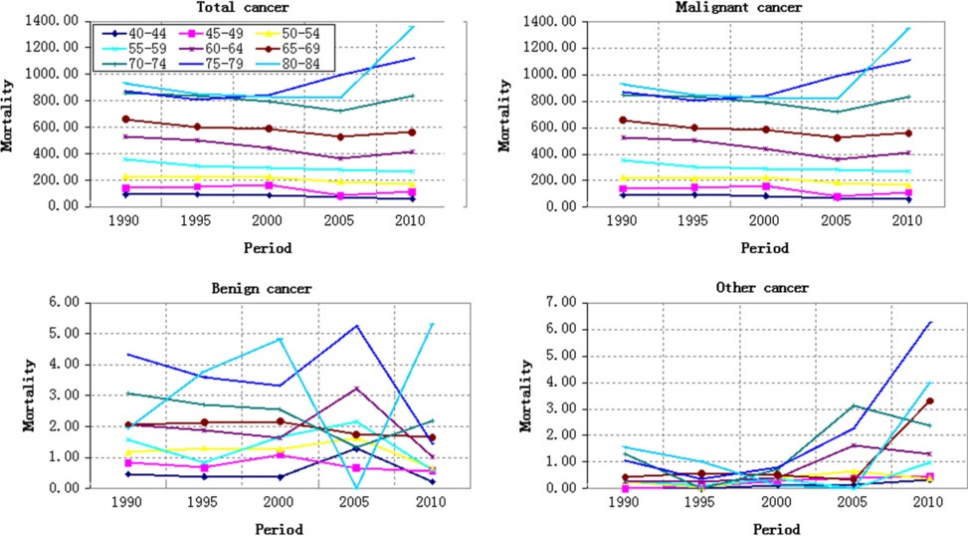
Cancer mortality (100,000 persons) variation of different age groups during 1990–2010: 40–84 years old.

The cancer mortality variation of different age groups from 1990 to 2010 was shown in Figures 2 and 3. Figure 2 showed the cancer mortality of 0–39 years old, while Figure 3 showed the cancer mortality of 40–84 years old. Except for Group 5–9 and Group 55–59 that decreasing continuously, the cancer mortality of rest age groups fluctuated differently. The comparative analysis on the cancer mortality of age groups in different years demonstrated that the cancer mortality risk of Group 80–84 and Group 75–79 increased significantly during 2005–2010 and 2000–2010 respectively, while that of rest age groups changed gently. The cancer mortality risk of Group 80–84 increased by 65.17% from 821.16/100,000 in 2005 to 845.02/100,000 in 2010, showing an annual average growth of 13.03%. The cancer mortality risk of Group 75–79 increased by 32.17% from 845.02/100,000 in 2000 to 1116.89/100,000 in 2010, showing an annual average growth of 3.22%. Viewed from the cancer mortality variation from 1990 to 2010, Group 0–4 increased slightly and Group 75–79 as well as Group 80–84 increased significantly, while the rest age groups decreased. The malignant cancer mortality wasn’t analyzed specially due to its similar variation law with that of total cancer mortality.

No obvious variation law of benign cancer mortality was observed in Figures 2 and 3. Different age groups exhibited different variation laws. However, the benign cancer mortality fluctuated more violently during 2000–2010 than that during 1990–2000. The benign cancer mortality risk showed a V-shaped variation trend from 2000 to 2010, while the benign cancer mortality risk was close to the X-axis from 1990 to 2000. Other cancer mortality risk changed gently during 1990–2000 but increased during 2000–2010.

#### Cohort-based variation of cancer mortality

**Figure 4:**
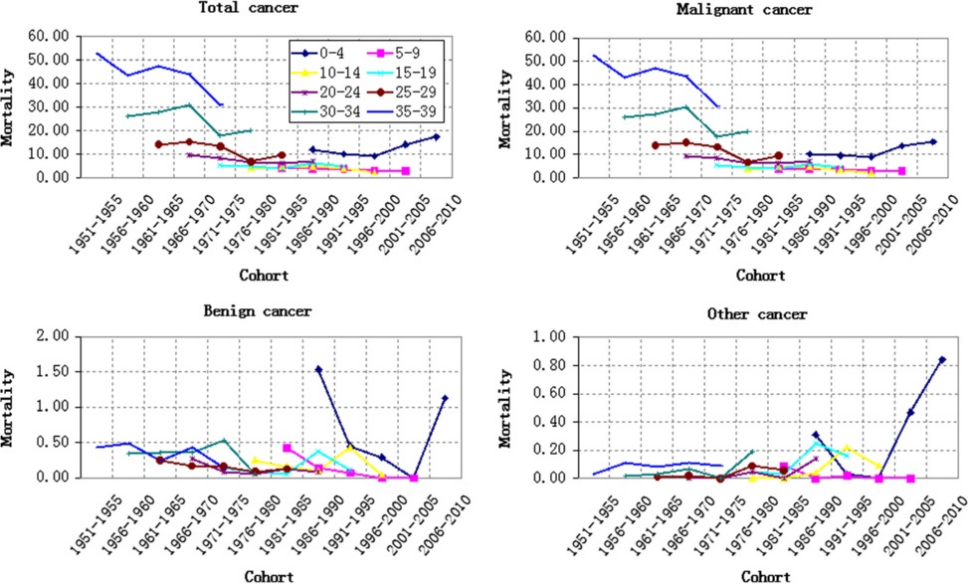
Cohort-based variation of age-specific cancer mortality (100,000 persons): 0–39 years old.

**Figure 5:**
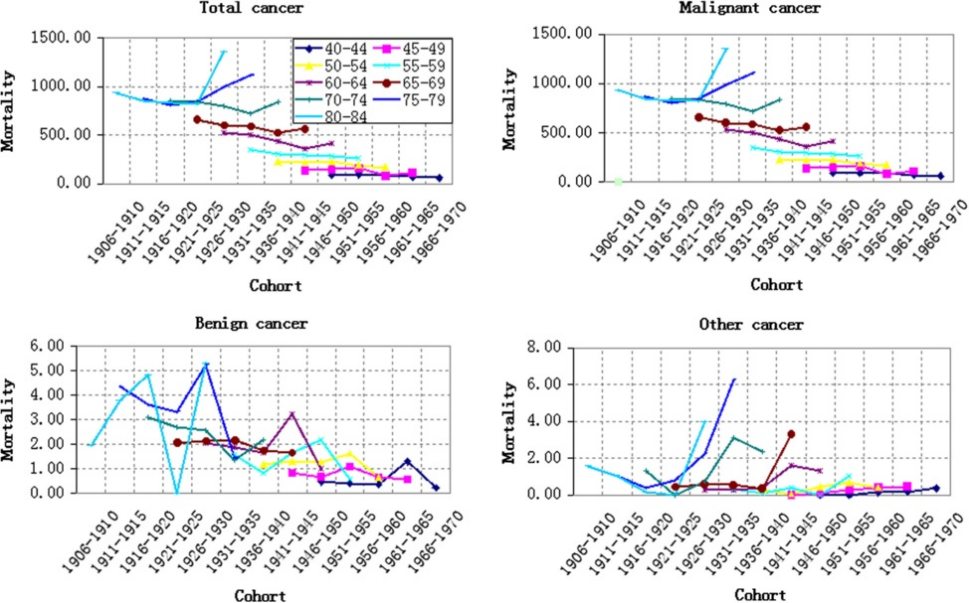
Cohort-based variation of age-specific cancer mortality (100,000 persons): 40–84 years old.

A cohort-based chart of age-specific mortality was drawn to make an intuitive evaluation of the birth cohort effect. The cohort-based variation of age-specific cancer mortality was shown in Figures 4 and 5. Different age groups showed different cohort-based variations of total cancer mortality. Except for Group 5–9 and Group 55–59 that decreasing continuously, the cancer mortality of all age groups fluctuated with the change of cohort year. Generally speaking, younger groups suffered less cancer mortality risks than older groups. The cohort-based variation of different types of cancer mortality will be discussed in the following text through APC model.

### APC model analysis results of cancer mortality

Age effect, period effect and cohort effect can be explored through APC model, thus enabling to study the impact of independent factors on the variation of cancer mortality. The APC model analysis results of age-specific cancer mortality were listed in Table 1.

**Table 1:** APC model analysis results of cancer mortality in rural China

	**Total cancer**		**Malignant cancer**		**Benign cancer**		**Other cancer**	
	**APC**	**(SE)**	**APC**	**(SE)**	**APC**	**(SE)**	**APC**	**(SE)**
**Intercept**	4.1765	0.0355	4.1558	0.0364	−0.7551	0.5315	−1.8801	0.4333
**Age(year)**								
0-4	−0.6590	0.1729	−0.7038	0.1791	0.5163	1.1350	0.6330	1.5291
5-9	−1.9272	0.2320	−1.9445	0.2377	−1.1271	1.4011	−1.9687	3.0150
10-14	−1.8556	0.2188	−1.8935	0.2264	−0.8411	1.1985	−0.5159	1.8210
15-19	−1.7353	0.1944	−1.7482	0.1996	−1.2748	1.2196	−0.6843	1.8427
20-24	−1.5254	0.1673	−1.5106	0.1700	−1.5462	1.2851	−1.9324	2.5174
25-29	−1.2398	0.1489	−1.2276	0.1511	−1.4669	1.2144	−1.4154	2.6771
30-34	−0.6662	0.1206	−0.6567	0.1224	−0.7488	0.9665	−0.9775	2.2841
35-39	−0.3162	0.1006	−0.3022	0.1021	−0.8108	0.9190	−0.2744	2.1731
40-44	0.0720	0.0820	0.0851	0.0831	−0.3713	0.7744	−0.3830	1.9106
45-49	0.4089	0.0660	0.4210	0.0668	−0.0609	0.6661	0.2427	1.5079
50-54	0.7334	0.0513	0.7428	0.0518	0.4817	0.5744	0.5105	1.2642
55-59	0.9497	0.0394	0.9590	0.0397	0.6508	0.5482	0.1627	1.0776
60-64	1.2226	0.0325	1.2295	0.0328	0.9617	0.5374	1.0716	0.7773
65-69	1.4060	0.0346	1.4117	0.0351	1.0818	0.6058	1.1048	0.6529
70-74	1.6549	0.0434	1.6587	0.0443	1.2643	0.6944	1.6847	0.6231
75-79	1.7405	0.0563	1.7417	0.0575	1.6493	0.7989	1.5872	0.7320
80-84	1.7367	0.0712	1.7375	0.0726	1.6420	0.9360	1.1546	0.9871
**Period(year)**								
1990	−0.0668	0.0350	−0.0699	0.0356	0.1702	0.3538	−0.5033	0.6949
1995	−0.0793	0.0211	−0.0798	0.0213	−0.0147	0.2571	−0.9005	0.5885
2000	−0.0537	0.0138	−0.0535	0.0139	0.0837	0.2102	−0.3285	0.4570
2005	−0.0387	0.0214	−0.0381	0.0216	0.0555	0.2543	0.4352	0.4313
2010	0.2385	0.0344	0.2414	0.0351	−0.2947	0.3709	1.2970	0.5957
**Cohort(year)**								
1906-1910	0.9928	0.0919	1.0120	0.0937	−0.3894	1.0242	1.6734	1.7152
1911-1915	0.9156	0.0735	0.9312	0.0750	0.4257	0.7101	1.1569	1.3716
1916-1920	0.9029	0.0574	0.9175	0.0586	0.4945	0.5565	0.4461	1.1632
1921-1925	0.9121	0.0427	0.9278	0.0436	−0.0499	0.4729	−0.4297	1.0864
1926-1930	0.9831	0.0298	0.9944	0.0305	0.6500	0.3163	0.6721	0.5993
1931-1935	0.8449	0.0257	0.8549	0.0262	0.1299	0.3769	0.8187	0.5010
1936-1940	0.6825	0.0319	0.6916	0.0324	0.2655	0.4417	−0.1999	0.7101
1941-1945	0.5315	0.0444	0.5348	0.0451	0.6363	0.5188	0.6714	0.8144
1946-1950	0.4604	0.0588	0.4647	0.0597	0.4620	0.6729	−0.2208	1.1755
1951-1955	0.3298	0.0747	0.3309	0.0759	0.4928	0.8198	0.4090	1.3562
1956-1960	−0.0161	0.0933	−0.0164	0.0947	0.3039	0.9907	−0.2930	1.6847
1961-1965	−0.0530	0.1096	−0.0585	0.1115	0.7783	1.0715	−0.3300	1.9534
1966-1970	−0.2125	0.1273	−0.2183	0.1294	0.4482	1.2565	−0.1442	2.1941
1971-1975	−0.5621	0.1517	−0.5684	0.1543	0.3477	1.3530	−1.8890	3.5877
1976-1980	−0.8006	0.1771	−0.8066	0.1802	−0.3217	1.6126	0.0147	2.1560
1981-1985	−0.8455	0.1953	−0.8619	0.1996	0.2432	1.4630	−0.4238	2.6424
1986-1990	−0.8396	0.1730	−0.8873	0.1800	0.3938	1.3209	0.5025	1.6030
1991-1995	−1.0576	0.2095	−1.0736	0.2164	−0.1679	1.5222	−0.2542	1.7865
1996-2000	−1.3019	0.2523	−1.2730	0.2569	−1.2491	2.0321	−1.8511	3.1430
2001-2005	−0.9667	0.2430	−0.9380	0.2476	−4.5496	8.7121	−0.1049	1.8721
2006-2010	−0.9002	0.2714	−0.9577	0.2861	0.6557	1.8177	−0.2243	1.7779
**Deviance**	95.7702		94.1017		12.1555		7.7004	
**DF**	45		45		45		45	
**AIC**	8.1011		8.0605		2.7343		1.9656	
**BIC**	−104.1491		−105.8176		−187.7638		−192.2189	

#### Age effect

**Figure 6:**
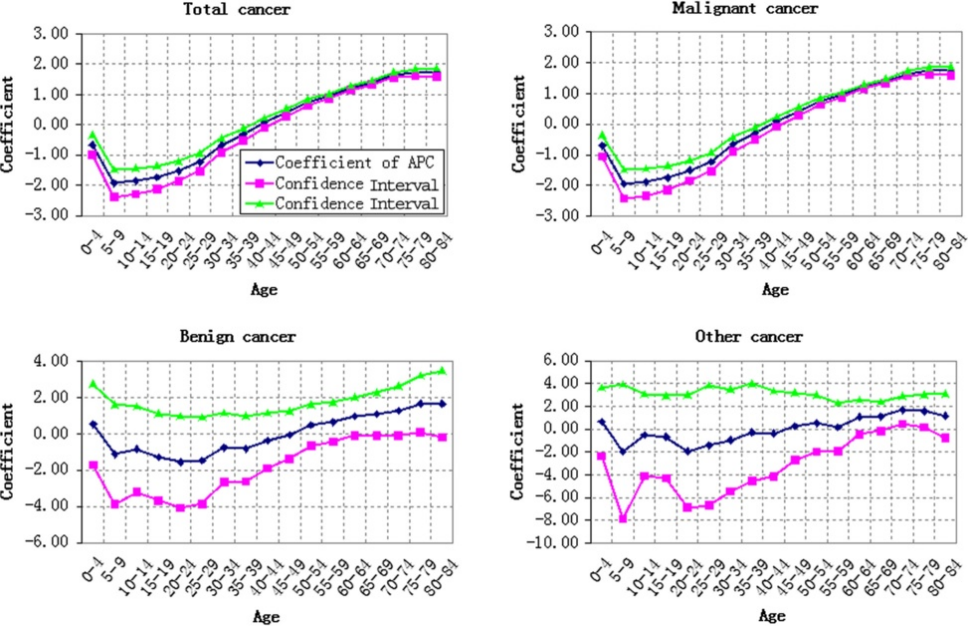
Age effect on cancer mortality in rural China.

In Table 1 and Figure 6, the coefficient of cancer mortality estimation showed a V-shaped variation trend with ages. Group 5–9 has the lowest cancer mortality. This coefficient decreased by 1.2682 from Group 0–4 to Group 5–9 and the corresponding mortality risk reduced by 71.87%. The mortality risk of Group 75–79 was more than 38 times than that of Group 5–9. From Group 5–9 to Group 75–79, the growth rate of mortality increased firstly and then decreased, reaching the peak at Group 30–35, 77.45% higher over the previous year. The mortality reduced from Group 75–79 to Group 80–84.

The benign cancer mortality of younger population decreased firstly and then increased. The minimum coefficient of age effect estimation was in Group 20–24. Other cancer mortality in rural China represented a wave increase with the growth of age.

#### Period effect

**Figure 7:**
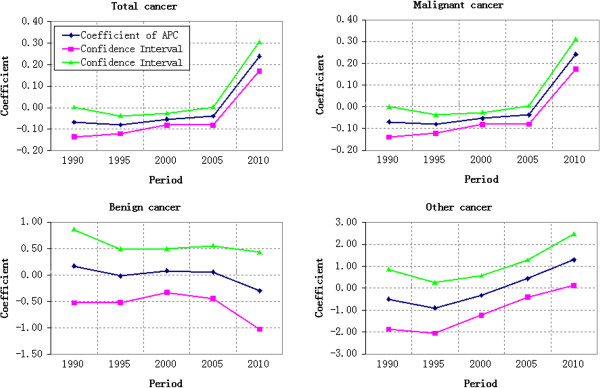
Period effect on cancer mortality in rural China.

The period effect on the total cancer mortality was estimated to increase according to Table 1 and Figure 7. The coefficient of mortality estimation achieved a net increase of 0.3053 = [0.2385-(−0.0668)] from 1990 to 2010. It indicated that the period effect alone increased the total cancer mortality risk by 35.70% from 1990 to 2010, showing an annual average growth of 1.79%. The total cancer mortality risk reduced by 1.24% from 1990 to 1995 and only increased by 4.15% from 1995 to 2005. However, the total cancer mortality risk increased significantly (31.94%) from 2005 to 2010.

The period effect on benign cancer mortality generally decreased. According to Table 1, the coefficient of estimation increased by 0.0984 from 1995 to 2000 and the corresponding mortality increased by 10.34%. However, such coefficient decreased by 0.4649 from 1990 to 2010 and the corresponding mortality risk reduced by 37.18%. The greatest decrease of mortality was occurred during 1990–1995 and 2005–2010. The coefficient of estimation of period effect on other cancer mortality changed significantly, which increased firstly and then decreased. It decreased by 0.3972 from 1990 to 1995 and then increased by 2.1975 from 1995 to 2010, showing a growth of more than eight times.

#### Cohort effect

**Figure 8:**
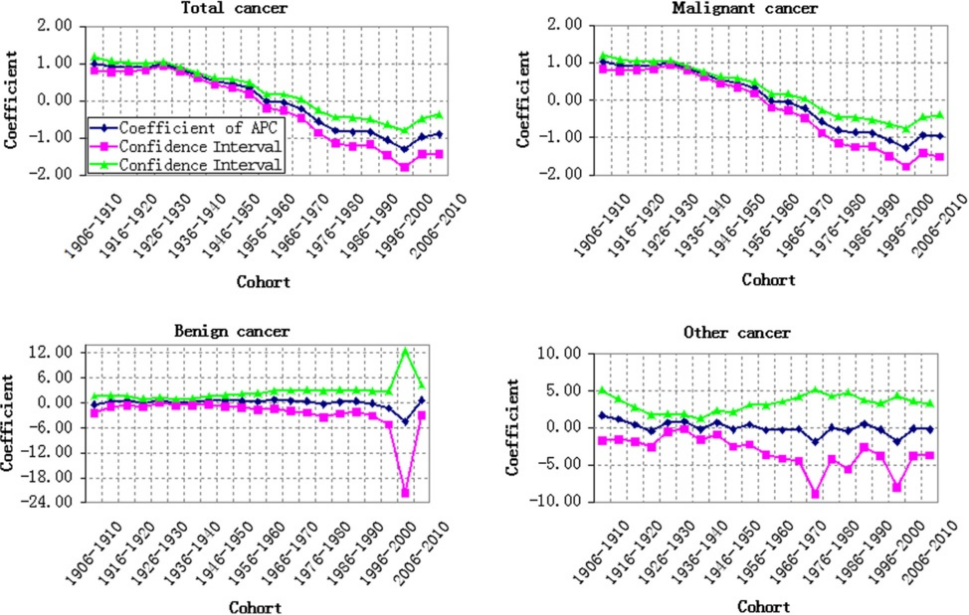
Cohort effect on cancer mortality in rural China.

The cohort effect on cancer mortality was shown in Figure 8. The total cancer mortality risk decreased by 84.94% from the birth year of 1906 to 2010. There were three rises of birth cohort effect from 1906 to 2010. The first rise was from 1916–1920 to 1926–1930, reaching the peak with a growth of 0.0802 (odds ratio = 1.0835). The second rise was from 1981–1985 to 1986–1990 with a growth of 0.0059 (odds ratio = 1.0059). The third rise started from 1996–2000 with a growth of 0.4017 (odds ratio = 1.4944) till 2006–2010.

The coefficient of estimation of birth cohort effect on benign cancer mortality achieved a great wave growth from 1906 to 2010. According to Table 1 and Figure 8, the mortality coefficient of population born during 2006–2010 was 1.0451 higher than that born during 1906–1910 and the corresponding cancer mortality risk almost doubled. It demonstrated a relative lower mortality coefficient (−4.5496) of population born during 2001–2005 (Table 1). This was caused by the lower cancer mortality risk of population born during 2001–2005 (Group 0–4 and Group 5–9 in 2005). The cohort effect on other cancer mortality risk fluctuated more violently compared with that of total cancer mortality. The population born during 1906–1910 expressed the highest cancer mortality risk with a coefficient of 1.6734. Additionally, two obvious low mortality coefficients were observed in population born in 1971–1975 (−1.8890) and 1996–2000 (−1.8511).

### Comparison of different estimated effects of APC model on cancer mortality

**Figure 9:**
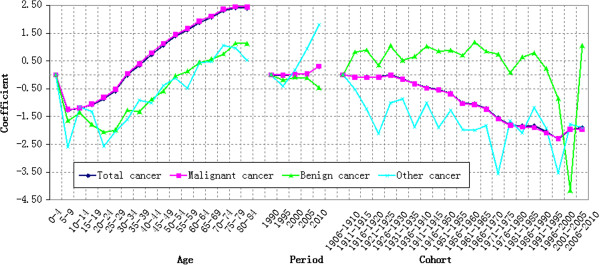
Estimated Age-Period-Cohort effects on cancer mortality from 1990–2010.

To make a more intuitive comparison of age, period and cohort effects estimated by APC model on different cancers, these three effects were converted to be initiated from 0. The converted age, period and cohort effects estimated by APC model from the total cancer mortality and different cancer mortalities were shown in Figure 9.

The age effect on the total cancer mortality decreased firstly and then increased, reaching the bottom at Group 5–9. The age effect on all of these three cancer mortalities represented a typical V-shaped trend. However, the coefficient of estimation of other cancer mortality fluctuated more violently, especially at younger groups. Furthermore, it decreased significantly after 70–74 years old. The estimation coefficient of benign cancer mortality also fluctuated at younger groups and increased continuously after 20–24 years old. The estimation coefficient of malignant cancer mortality decreased before nine years old and then increased stably until 79 years old.

The broken line of period effect on the total cancer mortality basically extended towards the horizontal direction until the significant increase of estimation coefficient in 2005. The malignant cancer mortality, benign cancer mortality and other cancer mortality had different variation laws. The broken line of malignant cancer mortality changed similarly with that of total cancer mortality. The coefficient estimation of benign cancer mortality increased continuously to get close to the broken line of total cancer mortality during 1990–2005, but began to decrease after 2005. The estimation coefficient of other cancer mortality decreased firstly and then increased, especially after 1995.

The cohort effect on the total cancer mortality risk decreased generally. However, a great gap of variation law was observed among the malignant cancer mortality, benign cancer mortality and other cancer mortality. With similar variation law of the total cancer mortality, the estimation coefficient of malignant cancer mortality decreased stably with a slight fluctuation. The estimation coefficient of benign cancer mortality showed a wave growth and its broken line maintained at the top position. However, the estimation coefficient of other cancer mortality represented a wave decrease and its broken line was in the bottom, crossing with that of malignant cancer mortality.

### Variation velocity analysis of cohort effect

**Figure 10:**
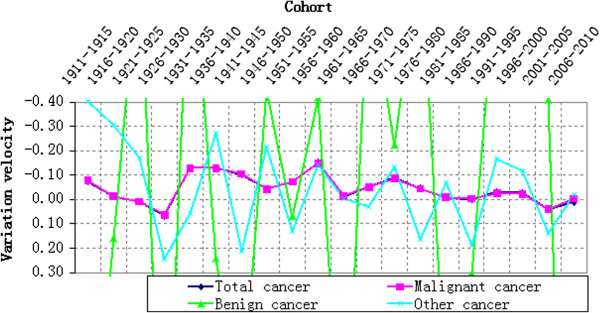
**Variation velocity of cohort effect on cancer mortality during 1911–2010.** Note: To clearly represent the variation law of cohort effect, the Y-axis of the statistical chart didn’t include all variation velocities of benign cancer mortality.

To have a more accurate understanding on the variation law of cohort effect, we converted the estimated effects into odds ratio by taking 1906–1910 as reference point. Next, the odds ratio of the year subtracted the odds ratio of previous year according to the principle of numerical differentiation, which shall be positive when mortality risk increased, negative when mortality risk reduced and 0 when mortality risk remained same. If the mortality risk reduced or increased at a constant speed, the corresponding line in Figure 10 will be parallel to the X-axis. Larger distance between the line and X-axis represents quicker variation of mortality risk, while shorter distance represents slower variation of mortality risk. Similarly, if the line distances away from the X-axis gradually, the variation velocity of mortality risk accelerates, otherwise, decelerates.

It can be seen from Figure 8 that the variation velocity of cohort effect on both of the total cancer mortality and different types of cancer mortality fluctuated during 1911–2010. For the total cancer mortality, most parts of its broken line were above the X-axis (negative). Just as stated above, the cohort effect decreased generally, which had three accelerating decreases. The first accelerating decrease started from 1926–1930 to 1936–1940. The second accelerating decrease started from 1946–1950 to 1956–1960, accelerated by 0.11 from −0.04 to −0.15. The third accelerating decrease started from 1961–1965 to 1971–1975, accelerated by 0.08 from −0.01 to −0.09. In Figure 10, the variation velocity of benign cancer mortality fluctuated violently without certain law. The variation velocity of other cancer mortality also fluctuated between above and below the X-axis, which further indicated the alternation of increase and decrease of cohort effects. The velocity of increase and decrease of benign cancer mortality varied in different birth years, thus resulting in no strong variation law.

## Conclusion

The age effect on the total cancer mortality shows a typical V-shaped variation trend. It decreases by 72% from Group 0–4 to Group 5–9 and then increases by more than 38 times until Group 75–79. It achieves the quickest increased at Group 30–35. Group 0–4 contributes high cancer mortality, which is certain related with the high incidence of childhood leukemia and embryonal malignant cancers before 5 years old. Viewed from the period effect during 1990–2010, the total cancer mortality decreased by 1.24% during 1990–1995 and increased only by 4.15% during 1995–2005. However, it increased significantly (32%) during 2005–2010. The Joinpoint analysis demonstrated that the total cancer mortality in United States kept stable during 1990–1993 but achieved an annual decrease of 1.1% during 1993–2002 and an annual decrease of 1.8% during 2002–2005 [26]. It indicates that China still has a long way to go to reduce its cancer incidence and mortality.

Viewed from the cohort effect during 1906–2010, the total cancer mortality decreased generally with four “deterioration periods” and three “improvement periods”. This is closely related with China’s social change in recent one hundred years. The first deterioration period (from 1916–1920 to 1926–1930) echoed with the first revolutionary war when suffered continuous wars, caused great damages to the medical and health services. Serious environmental destructions caused by wars also increased the environmental cancer risk. Such destructions lasted until 1930. The second deterioration period is from 1956-1960 to 1961-1965, coinciding with the three years of natural disasters. The third deterioration period (from 1981–1985 to 1986–1990) coincided with the reform and opening-up policy in 1978. During this period, China strived to accelerate economic development and industrialization, which brought serious air, water and soil pollutions, led to environmental cancer risk. During the fourth deterioration period (from 1996–2000 to 2006–2010), the progresses of urbanization, industrialization and globalization caused ecological environment deterioration, frequent occupational exposure and dietary structure as well as lifestyles change. These factors, together with aging of population and smoking, increased the cancer mortality continuously.

Although the above three “deterioration periods” attacked people’s health and social economic development, China still achieved outstanding fruits in health-care development in recent one hundred years and cancer prevention since 1950s [27]. Particularly, the total cancer mortality had three “improvement periods”. The first improvement period started from 1926–1930 to 1936–1940. This is beginning of medical and public health service in China. The public health in rural China achieved outstanding improvements, which facilitated the effective cancer prevention and increased cancer survivals [28]. The second improvement period (from 1946–1950 to 1956–1960) was benefited from the rehabilitation and national economic prosperity after liberation. After the founding of new China, the public health services implemented in rural in 1930s was maintained, and the health-center system of counties proposed by the Department of Health was recovered and re-established [28]. Furthermore, China’s basic environmental health conditions were improved effectively through “prevention-oriented” measures [29]. On the other hand, China initiated the cancer prevention since late 1950s. China determined six cancers (e.g. esophagus cancer and cervical cancer) as key prevented common cancers in 1958. National cervical cancer survey has been organizing since 1958. In 1959, the esophagus cancer was studied in four provinces and one city in North China and corresponding prevention stations were established [30]. The third improvement period (from 1961–1965 to 1971–1975) was the key period of cancer prevention in China. During this period, the National Cancer Prevention and Research Institute were founded; the first national survey on cause of death was accomplished; the distribution law and characteristics of malignant cancer mortality in China were disclosed. On this basis, a series of research institutes of cancer high-incidence areas and cancer prevention were established and a cancer prevention team was trained. These contributed a great stride of China’s cancer prevention during this period [27].

Statistical analysis on malignant, benign and other cancer mortalities as well as the total cancer mortality in rural China is carried out. The results demonstrate that malignant cancer mortality has similar variation law with the total cancer mortality, while benign and other cancer mortalities have unique variation features. Since the benign and other cancer mortalities account for less than 10% of the total cancer mortality, they won’t influence the variation law of the total cancer mortality. Finally, this paper also has some limitations. It involves neither gender-specific statistics of cancer mortality nor independent analysis of some harmful malignant cancers, such as lung cancer, liver cancer, stomach cancer, esophagus cancer, colorectal cancer, etc. However, a deep analysis on the variation of cohort effect on cancer mortality in rural China since 1906 is carried out in considering of China’s modern history, development of health service and cancer prevention. The research results are beneficial for predicting future trend of cancer mortality in China, exploring the influence factors of cancer incidence, and providing some references to domestic and foreign cancer prevention.

## Competing interests

The authors declare that they have no competing interests.

## Authors’ contributions

PW and CY participated in the conception and design of the study, data collection and statistical analysis, drafts of the manuscript. CX participated in the analysis of the literature, contributed to drafts of the manuscript. All authors read and approved the final manuscript.
